# Hospitalization for Oral Health-Related Conditions of the Australian Ageing Population: Two Decades of Analysis

**DOI:** 10.3390/geriatrics7010002

**Published:** 2021-12-22

**Authors:** Wisam Kamil, Estie Kruger, Berwin Turlach, Marc Tennant

**Affiliations:** 1 Department of Anatomy, Physiology and Human Biology, School of Human Sciences, The University of Western Australia, Perth, WA 6009, Australia; estie.kruger@uwa.edu.au (E.K.); marc.tennant@uwa.edu.au (M.T.); 2 Department of Mathematics and Statistics, School of Physics, Math and Computing, The University of Western Australia, Perth, WA 6009, Australia; berwin.turlach@uwa.edu.au

**Keywords:** hospitalization, ageing population, oral health conditions, separations, dental caries

## Abstract

The burden of oral health care increases among older people, with a profound challenge in utilising dental services in primary dental care settings. This study aimed to analyse two decades of nationwide hospital separation patterns due to oral health-related conditions among older people. Ageing population data were obtained from the Australian Bureau of Statistics, including people aged 65 years and older. All principal diagnoses of oral health conditions (ICD-10-AM) were analysed in this study. The hospitalisation data included all separations of older people for the financial years 1998–1999 to 2018–2019. A total of 205,461 hospital separations were recorded for older people over a period of twenty-one years. More than 60% of these separations were collectively attributed to dental caries, disorders of teeth and supporting structures, diseases of the jaws, diseases of the pulp and periapical tissues. However, the average rate of separations per 10,000 people due to dental caries was the highest among the dental conditions (8.68). Furthermore, the remaining oral health-related conditions predict an annual percentage increase in the rate that would compromise their oral health quality of life. Dental caries and its sequela seem to be the leading cause for oral health-related hospital admissions in Australia for people aged 65 and older. This could be an indicator of the inadequacy of disease management in the primary dental care setting.

## 1. Introduction

The world is facing an ageing demographic transition where older people are projected to outnumber children [1]. This situation is accompanied by increased demands for ageing in place [2]. As a result, this generates further growing demands for health care services [3]. Moreover, the oral health needs of the ageing cohort have changed over the past two decades; and with more dentate individuals, oral health needs of the elderly have increased [4]. With respect to the chronic nature of oral diseases, there is an increased prevalence of tooth morbidity among the ageing population due to cumulative exposure to these diseases [5]. In addition, older people’s level of frailty and lack of dexterity further compromise their oral health and complicate the self-plaque control measures required for disease control and oral health stability, leading to an increase in periodontal diseases and root caries susceptibility [6]. Cohort studies identified the older population as an active carious lesion group, at a rate similar to adolescents and younger adults [7,8,9]. Unlike youngsters, older people have an increased prevalence of multiple systemic conditions, impairment, and use of medications [10,11], all impacting their oral health systems [4]. For example, the continuous exposure of medically compromised older patients to polypharmacy is a common factor influencing their salivary flow rate and buffering capacity associated with a high risk of dental caries [12]. Accordingly, physiologic and pathologic age changes in this population add an extra burden on the need for dental care services of elderly patients [12,13]. However, the clinical experience of the general dental workforce shows limited involvement in geriatric dentistry [14]. Studies identified the ineffectiveness of primary care as one of the factors that increase the number of preventable dental hospital admissions [15,16,17]. Hospital admissions due to oral health conditions are frequent among medically compromised patients and those with acute dental conditions [16]. However, most research studies reported only data on preventable dental hospital admissions [16,18,19]. A detailed analysis of the national hospitalisation data due to all oral health-related conditions among the older population has not been reported yet. A few studies highlighted the burden of hospitalisation on the health system due to oral health conditions among the ageing population, but that was only in Western Australia [18,20]. In addition, the concentration of this cohort varies across the states and territories [13]; therefore, this study aims to analyse in detail two decades of nationwide hospital separation patterns due to oral diseases among the geriatric population in Australia.

## 2. Materials and Methods

### 2.1. Ethics

The study involved a de-identified analysis of data that were accessed from open access websites; therefore, an exemption from the ethics review (RA/4/20/6185) was obtained from the Human Research Ethics Office at the University of Western Australia.

### 2.2. Population Data

The study population were obtained from the Australian Census of Population and Housing, collected by the Australian Bureau of Statistics. The study population included only people aged 65 years and older. This represents the entire ageing population using census data; therefore, no sampling was included in this study. Instead, all persons in the population were included; therefore, the sampling error is irrelevant [21].

### 2.3. Hospitalisation Data

Hospitalisation data were obtained from the Australian Institute of Health and Welfare for the twenty-one financial years 1998–1999 to 2018–2019 [22]. All principal diagnoses of oral health conditions (ICD-10-AM) were analysed in this study. Analysis was based on hospital separations of all older people, recorded by every private and public hospital in Australia for the study period. “Separation is the term used to refer to the episode of care, which can be a total hospital stay or a portion of a hospital stay beginning or ending in a change of type of care” [23]. The ICD-10-AM is the International Statistical Classification of Diseases and Related Health Problems, Tenth Revision, Australian Modification [24]. It is the standard classification system used for reporting diagnosis for diseases and other health problems for admitted patients across Australia hospitalisation services [24]. The principal diagnoses categories of oral health conditions K00–K14 “Diseases of oral cavity, salivary glands and jaws” included the following: K00 “Disorders of tooth development and eruption”, K01 “Embedded and impacted teeth”, K02 “Dental caries”, K03 “Other diseases of hard tissues of teeth”, K04 “Diseases of pulp and periapical tissues”, K05 “Gingivitis and periodontal diseases”, K06 “Other disorders of gingiva and edentulous alveolar ridge”, K07 “Dentofacial anomalies (including malocclusion)”, K08 “Other disorders of teeth and supporting structures”, K09 “Cysts of oral region, not elsewhere classified”, K10 “Other diseases of jaws”, K11 “Diseases of salivary glands”, K12 “Stomatitis and related lesions”, K13 “Other diseases of lip and oral mucosa”, and K14 “Diseases of tongue”.

### 2.4. Data Analysis

Descriptive data were analysed using Microsoft^®^ Excel for Mac (version 16.54, Microsoft, Seattle, WA, USA). The statistical analysis of the data was performed using R software, version 4.1.1 (R Foundation for Statistical Computing, Vienna, Austria). Separation rates for oral health conditions were calculated as the total number of separations for admitted older patients aged 65 and above divided by the total number of older people (≥65-year-old) in the population. It was presented as a rate per 10,000 individuals of older population [23].

### 2.5. Basic Model

The model consisted of various conditions for the years $t_{1}=1998,\ldots ,t_{21}=2018$ the number of cases $y_{i}$ in that year and the number of people at risk ($E_{i}$). In most cases the data seem to be overdispersed and we modeled it using the negative binomial distribution, but in some cases the Poisson distribution is sufficient. The models assume that the mean response $\mu_{i}$ is given by $r_{i}E_{i}$, i=1,…,21 and we modeled the logarithm of the rate $r_{i}$ as a polynomial in year with the number of people at risk as an offset, i.e., $\log\left(r_{i}\right)=\beta_{0}+\beta_{1}t_{i}+\cdots +\beta_{d}t_{i}^{d}$ The degree of the polynomial was by forward selection. If the chosen degree is d=1, then the coefficient $\beta_{1}$ has the interpretation that the rate at which the conditions occur in the population increases by a factor of $e^{\beta_{1}}$ from year to year. In other words, the rate increases by $100\left(e^{\beta_{1}}-1\right)\%$ from year to year.

## 3. Results

### 3.1. Descriptive Analysis

**Figure 1:**
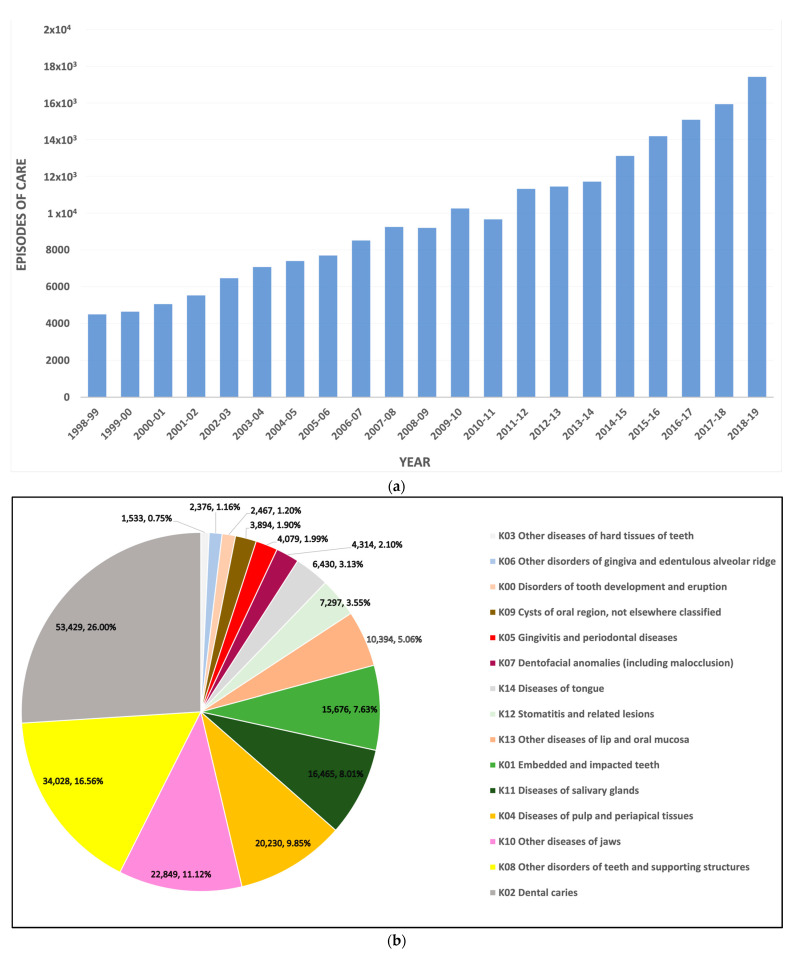
Descriptive analysis of hospital separations for oral health-related conditions among older people in Australia, (**a**) total separation numbers over a 21-year period, (**b**) total numbers and percentages by the diseases of oral cavity, salivary glands, and jaws.

There were 205,461 hospital separations between 1998 and 2019 due to oral health-related conditions among people aged 65 and older in private and public hospitals in Australia (Figure 1a). Overall, the average percentage of total separations tends to decrease with age-group increase. However, there was almost no difference in the average hospitalisation percentages over two decades with respect to gender (49.39% and 50.61% for males and females, respectively) [22].

Dental caries was the most common diagnosis attributed to these hospital separations (26%). The other leading diseases for entries were “diseases of the pulp and periapical tissues”, “diseases of the jaws”, and “disorders of teeth and supporting structures”, which accounted for 9.85%, 11.12%, and 16.56%, respectively (Figure 1b). However, each of the remaining cases contributed to less than 10% of the hospitalisation episodes, with the lowest value recorded for “other diseases of hard tissues of teeth” (0.75%). There was a noticeable gradual increase in the number of separations for a period of twenty-one years with slight fluctuation between 2008 and 2011 (Figure 1a). This fluctuation was mainly due to a discrepancy in the numbers of separations accounted for dental caries and diseases of the jaws.

### 3.2. Separation Rate

**Figure 2:**
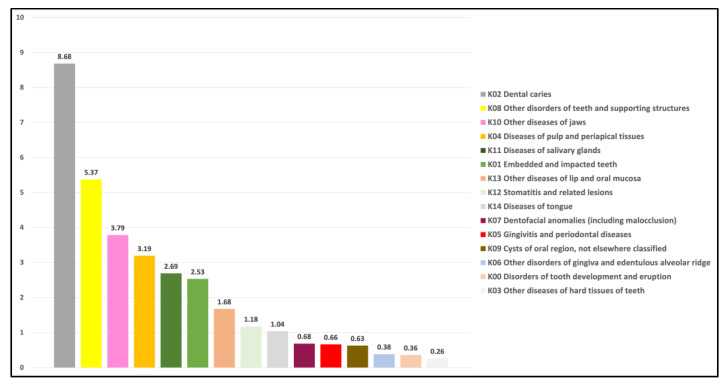
The average rate of episodes of separation per 10,000 of older population (≥65).

**Figure 3:**
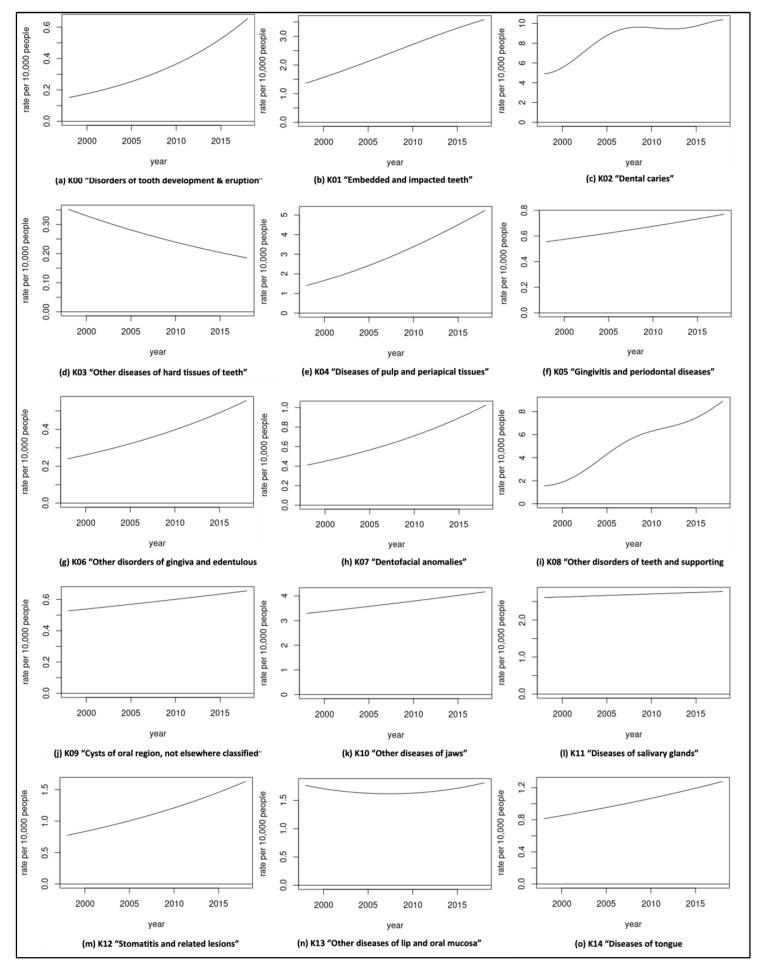
Separation rates (per 10,000) over two decades of older population (≥65).

**Figure 4:**
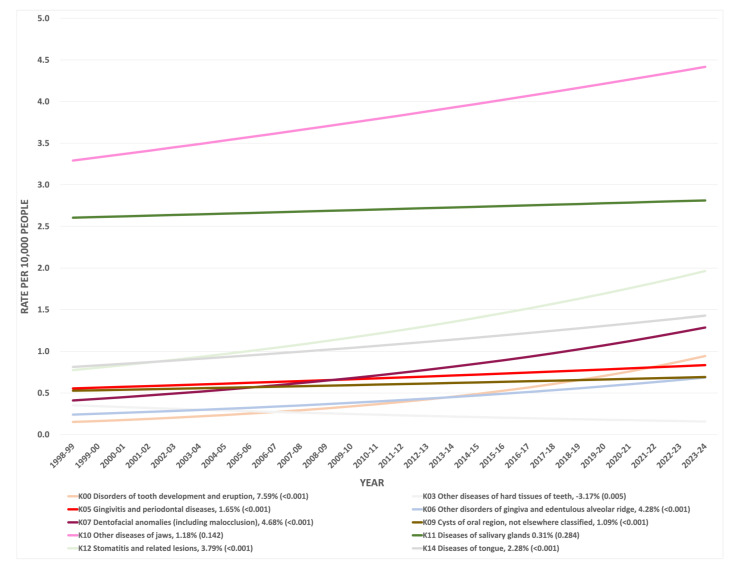
Predicted hospitalisation rates of older population (≥65) for years 1998–2023 for selected oral health conditions, including percentage of rate increase each year (*p*-value).

The rate of dental caries-related separations reached a peak of 10.43 (per 10,000 people) in the year 2018–2019 (Supplementary Figure S1c: The crude rates per 10,000 and 95% confidence intervals). Over two decades, the separation rate due to dental caries was the highest of all oral health conditions, with an average rate of 8.68, followed by the disorders of teeth and supporting structures (5.37) (Figure 2). The curve of caries-related separation rates has almost flattened since 2006; however, it had shown a slight increase again in 2018 (Figure 3c). In contrast, throughout the period of twenty years, the hospital separations with the diagnosis of diseases of the hard tissues indicated the lowest average rate of all conditions (0.26) (Figure 2). This is the only dental condition with an annual decrease of −3.17% in the rate over the two decades (Figure 4). On the other hand, the diseases of jaws revealed inconsistency in the separation rates for the duration of twenty years (Supplementary Figure S1k: The crude rates per 10,000 and 95% confidence intervals). This discrepancy rate comprised a notable rise in each of the following years, 2003, 2007, 2009, and 2011, accompanied by a marked decrease in 2008, 2010, 2013, and 2016. Regarding hospital separations due to diseases of the pulp and periapical tissues and embedded and impacted teeth, there was almost a steady increase in the rate between 1998 and 2019 (Supplementary Figure S1b,e: The crude rates per 10,000 and 95% confidence intervals) with an average rate of 3.19 and 2.53, respectively (Figure 2). In comparison, diseases of salivary glands separations contributed to an average rate of 2.69 (Figure 2) for the twenty years, with slight fluctuations that remained between 1998 and 2018 for 2.9 and 3.0, respectively (Supplementary Figure S1l: The crude rates per 10,000 and 95% confidence intervals), while the remaining oral-health related conditions had a rate of hospital separations less than 2 per 10,000 of older people (Figure 2).

### 3.3. Predicted Rate Increase

The predicted hospitalisation rates of the older population for the years 1998–2023 indicated a significant annual percentage increase in the rate of the following oral conditions: K00 “Disorders of tooth development and eruption”, K05 “Gingivitis and periodontal diseases”, K06 “Other disorders of gingiva and edentulous alveolar ridge”, K07 “Dentofacial anomalies”, K09 “Cysts of oral region”, K12 “Stomatitis and related lesions”, and K14 “Diseases of tongue”. The disorders of tooth development and eruption have the highest annual percentage increase (7.59%). On the other hand, other diseases of the hard tissues of teeth show an annual percentage decrease in the rate of 3.17%.

## 4. Discussion

This is the first study analysing two decades of nationwide hospital separation patterns due to oral health-related conditions among people aged 65 and older. Over this period, the number of separations, as well as the rate per a population of 10,000, in this ageing cohort increased consistently. Dental caries and its sequela seem to be the leading cause for oral health-related hospital admissions in Australia for people aged 65 and older. This could be an indicator of the inadequacy of disease management in the primary dental care setting [20]. Epidemiological and oral health reports confirm the reduction in complete tooth loss, and increased tooth retention amongst the ageing population, thus contributing to an increased prevalence of coronal and root caries [25,26]. This analysis of dental caries admissions shows a comparable percentage to the hospital separations of the age group (65–74) in the 10-year analysis study previously conducted in Western Australia [18]. Regarding separation rates due to dental caries, the steep increase in the fitted rate of hospitalisation due to this dental condition (from 1998–2004) supports the findings of other research, which found an accelerated annual coronal and root caries rate among older communities [7]. Cost barriers to access care in the private sector [27] and the current primary dental workforce not being fully prepared to respond to the oral health care needs of an ageing population [28], might also be contributing factors in the observed increase in dental caries-related hospitalisations.

Although most previous studies have focused on dental caries as a leading cause of hospital separations [29,30,31,32], two studies revealed more frequent admissions due to item code “disorders of teeth and supporting structures” among older people [18,20]. The results of our study identified ‘disorders of teeth and supporting structures’ as the second highest cause of admissions. This item code includes exfoliation of teeth due to systemic causes, loss of teeth due to accident, extraction or local periodontal disease, atrophy of edentulous alveolar ridge, and retained dental root. Disorders of teeth and supporting structures also showed a sharp increase in the fitted rate of hospitalisations from 1998–2004. Despite less edentulism among older people, the coexistence of chronic systemic diseases in older individuals is more likely to result in tooth loss [33]. In addition, regular denture wearing by the edentulous older people contributes to an increase in the prevalence of atrophic alveolar ridges [34]. The rate of these disorders was five times higher in 2018 than twenty years ago. This may be a reflection of insufficient geriatric dental care in the primary oral health care system [35].

Just two published studies reported on hospital separations due to diseases of the pulp and periapical tissues in older people [18,20], with one of these reporting similar percentages of admissions as in our study (10%) [18]. A combination of physiologic changes associated with ageing and pathologic pulpal changes associated with a high prevalence of root caries in older people would present practical challenges for the general dentist in endodontics treatment [4,36]. The calcific changes of the pulp-dentine complex, combined with old age frailty and dependency concerns, would sophisticate access and treatment of calcified root canals by non-specialist dentists; consequently, the high cost of specialist endodontics treatment may contribute to the increased prevalence of hospitalisations due to pulpal diseases among older people [36]. However, our analysis demonstrated a constant slower increase in the rate of these diseases than dental caries, and disorders of teeth and supporting structures; this could be interpreted as a result of adopting more preventive approaches during the past two decades, where more people had access to preventive and topical fluoride application visits [37,38].

Due to distinct fluctuation in separation rates for diseases of the jaws, we extended the analysis of the ICD-10-AM to include aggregate counts for this principal diagnosis at the 4-digit diagnosis level. Interestingly, the K10.2 “inflammatory conditions of jaws” accounted for 88% of total admissions attributed to diseases of jaws by older people over the age of 65 in 2003, 2007, 2009, and 2011. Inflammatory conditions of jaws include dentoalveolar and jaw infections with varying degrees of soft tissue involvement [39]. These diseases increase the susceptibility to life-threatening conditions due to the spreading of infections in medically compromised older people [40]. Therefore, the recurrent abrupt rise of admissions, mainly due to these age-associated inflammatory lesions of jaws, is serious enough to warrant the oral health care system to prioritise older patients’ primary dental care strategies. This could also be predicted based on the annual rate increase in the diseases of the jaws that is projected to reach 4.42 per 10,000 of older people by 2023.

Although most separations for embedded and impacted tooth removal were recorded for young patients [41]; the average rate of hospital separations for older people in our study was 2.53 per 10,000. The comorbidities and delay of healing postoperative surgical extractions among the ageing population may increase the risk of possible complications of impacted tooth removal [42]. Therefore, it is expected to observe this recorded rate in our analysis among the ageing cohort; however, the increased rate is slower than other leading diseases, which could be dictated by the decision of extraction at early age groups.

Although the remaining separations (for other conditions, including item codes K00, K03, K05, K06, K07, K09, K12, and K14) had a rate lower than 2 per 10,000 older people, a further increased pressure on the health system is expected based on the projected 5-year increase in the rate of hospitalisation for these conditions. In addition, the annual increase in these conditions may affect the overall oral quality of life among older people. Moreover, diseases that affect salivary flow would also compromise their oral health quality of life [43]. Although the annual rate increase in separations due to diseases of salivary glands account for only 0.3%, dry mouth increases the caries risk in older people, where hyposalivation is associated with systemic diseases and medications [4]. However, one of the limitations in this study is that no further analysis was conducted at the 4-digit diagnosis level that could expose particular diagnoses of these oral health conditions. In addition, this might explain the difficulty in commenting about the lowest rate observed due to separations for “other diseases of hard tissues of teeth”, where notable variations were observed at the broad scope of 4-digit diagnosis level nomenclature. Therefore, an adaptation of future research analysis in these specific aspects of the ICD-10-AM is suggested.

The literature persistently measures the effectiveness of primary interventions to reduce the demands on potentially preventable hospitalization [15,17]. However, the recorded hospitalisation growth in our study could be an indicator of a lack of timely and appropriate intervention in the primary care sector. A recent study by Kamil et al. also reported an uneven spatial distribution of dental practices in relation to the ageing population, resulting in access issues for some [44]. Increasing rates of dental hospitalisations among older people could also be an indicator of lack of skills and knowledge in geriatric dentistry among the dental workforces. This would reflect the lack of recognition of geriatric dentistry in the Australian dental curriculum [28]. The inadequacy in the geriatric dental pathway has remained an issue for decades, despite significant barriers older people encounter to access dental services [35]. Additionally, recent geriatric oral health research highlighted the maldistribution of this cohort across Australia on the utilisation of dental services [13]. This variation in the utilisation of the services by states and territories could influence the hospitalisation rates where older people are concentrated. However, one of the limitations of this study is the lack of individual-level demographic data, which would enable identification of geographical and socio-economic indicators of hospitalisation in this cohort. In addition, the unprecedented period after March 2020 would affect our predicted projections as hospital activities were affected by the COVID-19 pandemic, along with other factors that substantially affected their morbidity and mortalities [45].

## 5. Conclusions

The ageing cohort separation data analysis indicated an apparent increase in the hospitalisation rate due to oral health-related conditions over twenty-one years. Despite the prevention-based efforts to reduce the risk of dental caries, it is the primary dental health condition attributed to oral health-related hospitalisations in the older people of Australia. This persistent increase in the separation rate may reflect a quiet crisis in geriatric dentistry, indicating an insufficiency of primary dental care interventions. Moreover, comorbidity in older people may inflate the burden on the hospitalisation oral health system of the elderly in Australia.

## Data Availability

The data were accessed from publicly available open-access websites; therefore, data availability sharing is not applicable.

## Supplementary Materials

The following are available online at https://www.mdpi.com/article/10.3390/geriatrics7010002/s1, Figure S1, The crude rates per 10,000 people (95% confidence intervals) for separations of older people in Australia by the diseases of oral cavity, salivary glands, and jaws (a–o).
