# Supplementary material for: Hospitalization for Oral Health-Related Conditions of the Australian Ageing Population: Two Decades of Analysis
Source: Geriatrics (Basel). 2021 Dec 22;7(1):2. doi: 10.3390/geriatrics7010002 (PMC8788296; doi:10.3390/geriatrics7010002)
Supplement: Supplementary file 1 [file geriatrics-07-00002-s001.zip › geriatrics-1496452-supplementary.pdf]

Figure S1, The crude rates per 10,000 people (95% confidence intervals) for separations of older people in Australia by the diseases of oral cavity, salivary glands and jaws (a-o). vertical (y) axis represents the financial years 1998-1999 to 2018-2019 and horizontal (x) axis represents the separation rate together with 95% confidence intervals.

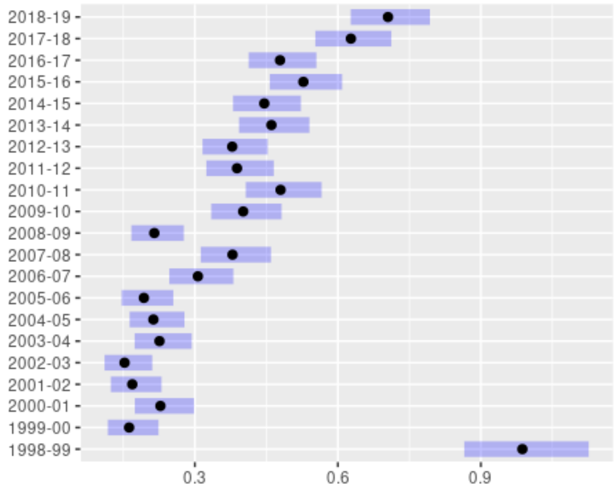

(a) K00 “Disorders of tooth development and eruption”

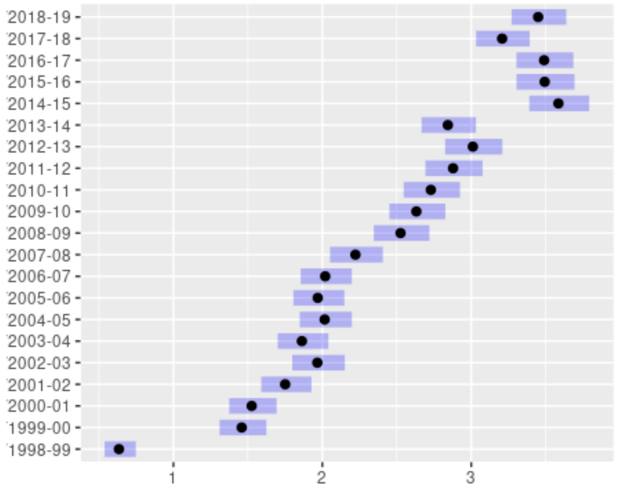

(b) K01 “Embedded and impacted teeth”

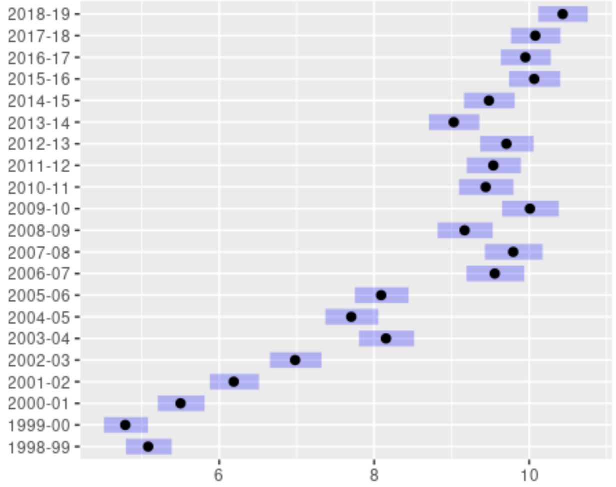

(c) K02 “Dental caries”

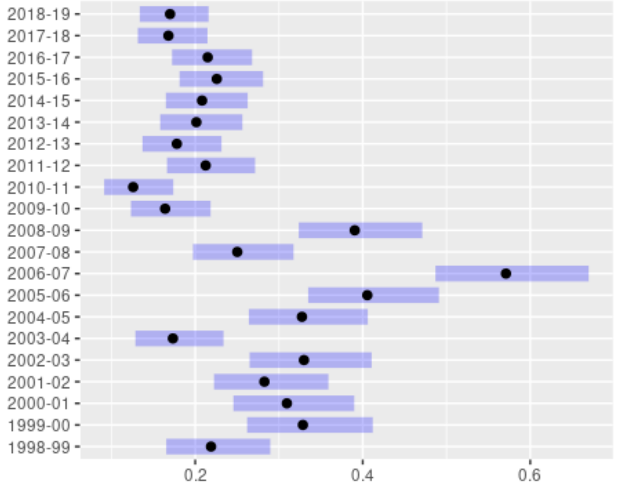

(d) K03 “Other diseases of hard tissues of teeth”

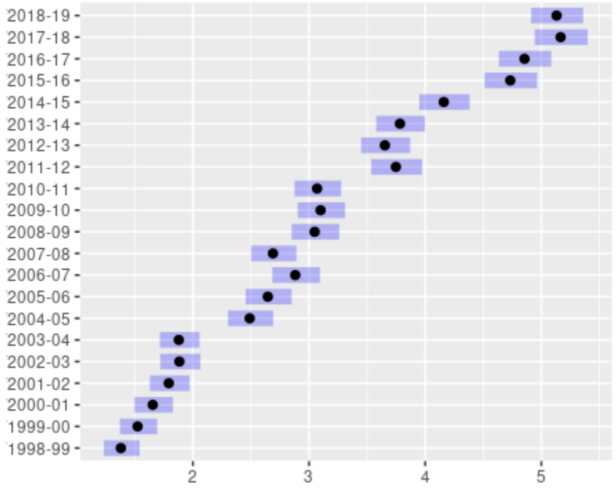

(e) K04 “Diseases of pulp and periapical tissues”

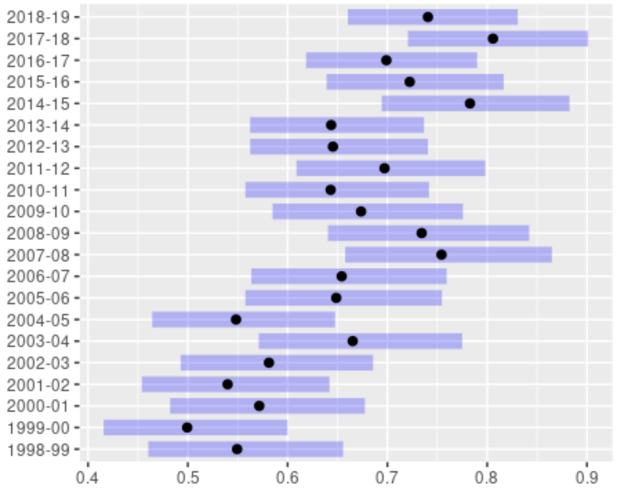

(f) K05 “Gingivitis and periodontal diseases”

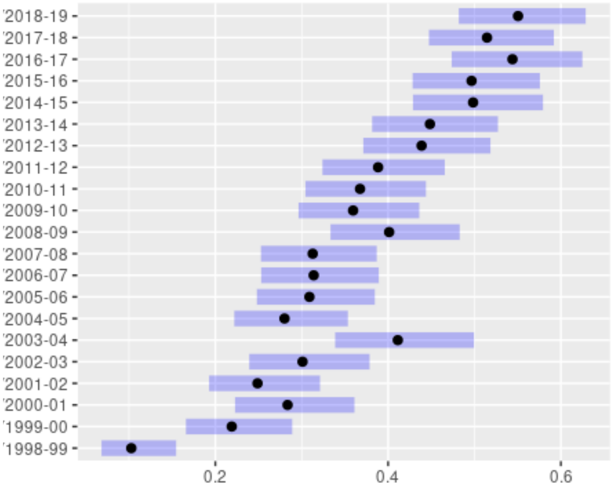

(g) K06 “Other disorders of gingiva and edentulous alveolar ridge”

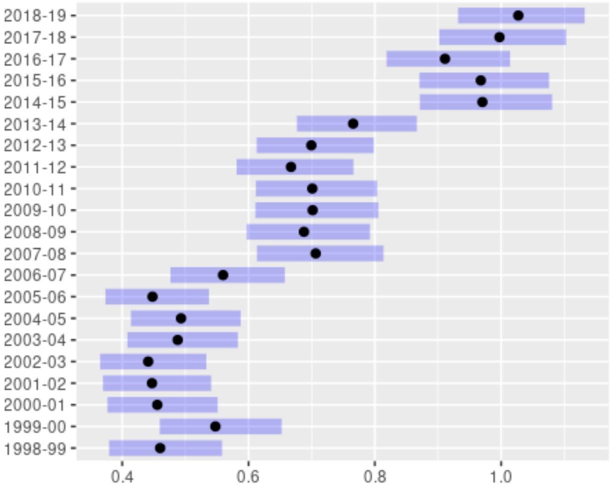

(h) K07 “Dentofacial anomalies (including malocclusion)”

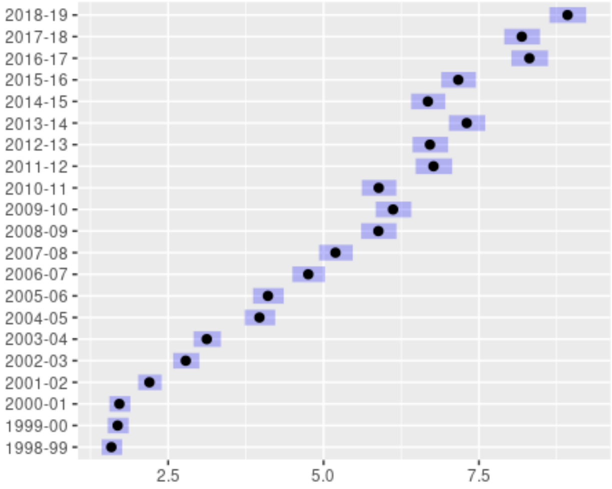

(i) K08 “Other disorders of teeth and supporting structures”

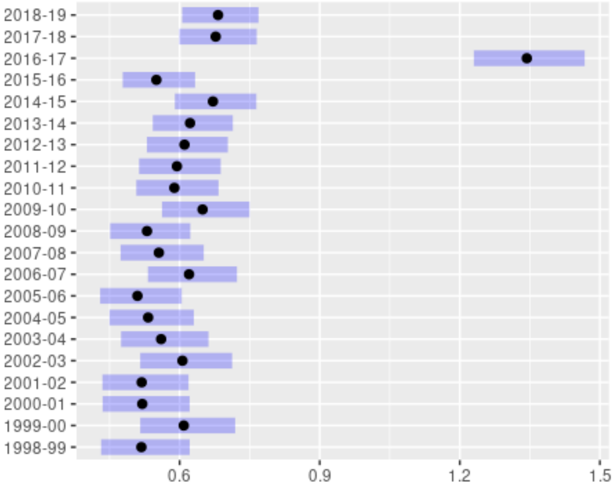

(j) K09 “Cysts of oral region, not elsewhere classified”

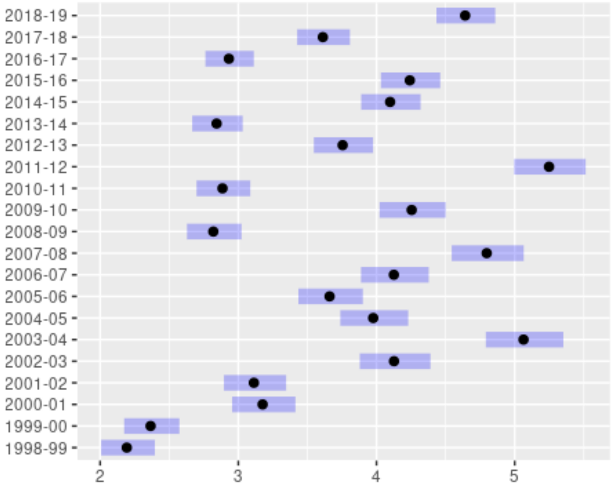

(k) K10 “Other diseases of jaws”

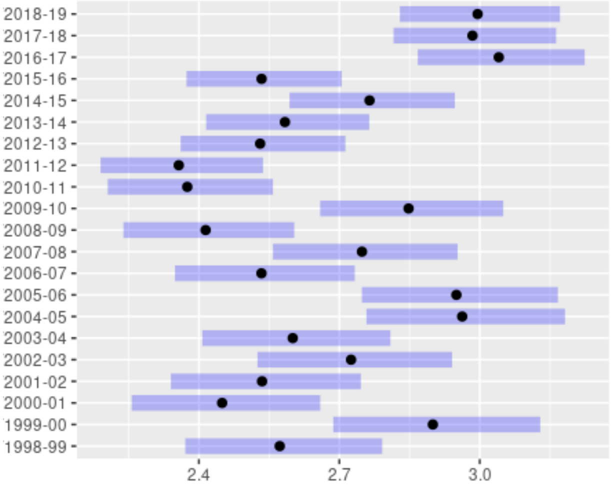

(l) K11 “Diseases of salivary glands”

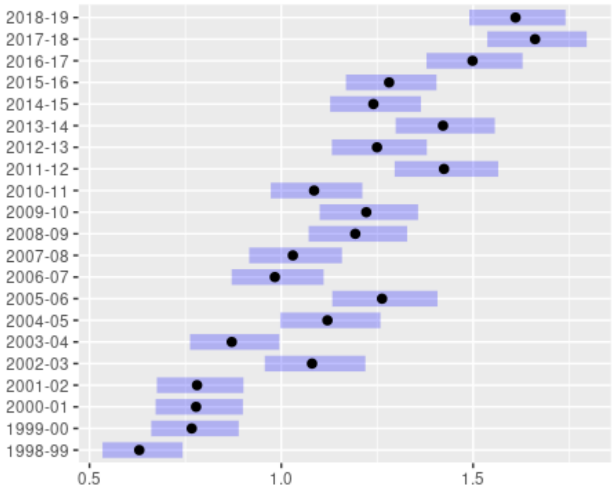

(m) K12 “Stomatitis and related lesions”

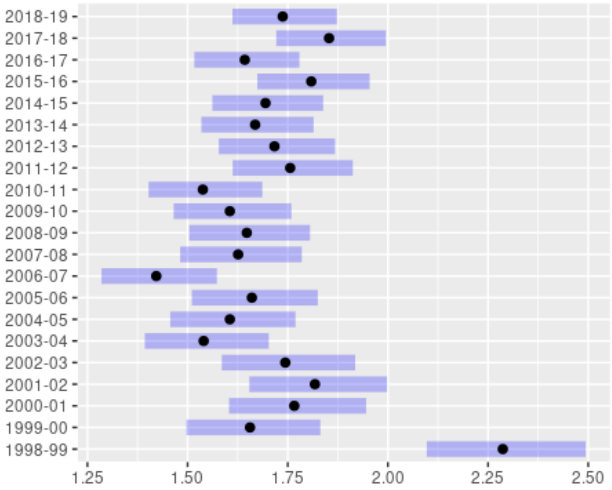

(n) K13 “Other diseases of lip and oral mucosa”

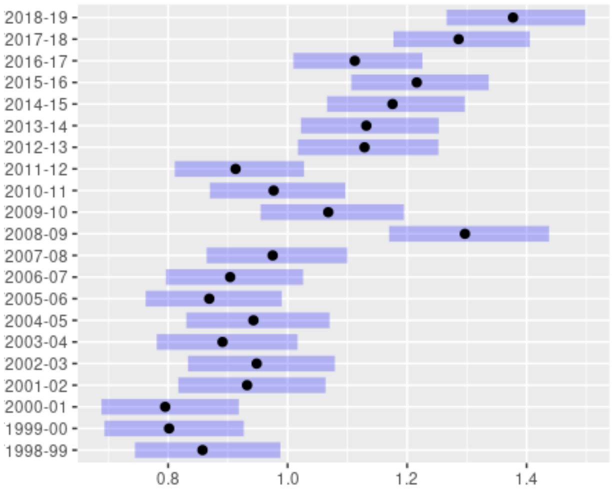

(o) K14 “Diseases of tongue
